# The Role of 18F-FDG PET/CT in Guiding Precision Medicine for Invasive Bladder Carcinoma

**DOI:** 10.3389/fonc.2020.565086

**Published:** 2020-10-06

**Authors:** Antoine Girard, Helena Vila Reyes, Hiram Shaish, Jean-François Grellier, Laurent Dercle, Pierre-Yves Salaün, Olivier Delcroix, Mathieu Rouanne

**Affiliations:** ^1^Department of Nuclear Medicine, Centre Eugène Marquis, Université Rennes 1, Rennes, France; ^2^Department of Urology, Columbia University Irving Medical Center – New York Presbyterian Hospital, New York, NY, United States; ^3^Department of Radiology, Columbia University Medical Center, New York, NY, United States; ^4^Centre Cardiologique du Nord, Nuclear Medicine, Saint-Denis, France; ^5^Department of Radiology, New York Presbyterian Hospital – Columbia University Medical Center, New York, NY, United States; ^6^Department of Nuclear Medicine, Centre Hospitalier Régional Universitaire de Brest, Brest cedex, France; ^7^Department of Urology, Hôpital Foch, Université Versailles-Saint-Quentin-en-Yvelines, Université Paris-Saclay, Suresnes, France

**Keywords:** PET – Positron Emission Tomography, bladder cancer, muscle invasive bladder cancer, immunotherapy, staging

## Abstract

Bladder cancer (BC) is the 10th most common cancer worldwide. Approximately one quarter of patients with BC have muscle-invasive disease (MIBC). Muscle-invasive disease carries a poor prognosis and choosing the optimal treatment option is critical to improve patients’ outcomes. Ongoing research supports the role of 2-deoxy-2-(18F)fluoro-D-glucose positron emission tomography (18F-FDG PET) in guiding patient-specific management decisions throughout the course of MIBC. As an imaging modality, 18F-FDG PET is acquired simultaneously with either computed tomography (CT) or MRI to offer a hybrid approach combining anatomical and metabolic information that complement each other. At initial staging, 18F-FDG PET/CT enhances the detection of extravesical disease, particularly in patients classified as oligometastatic by conventional imaging. 18F-FDG PET/CT has value in monitoring response to neoadjuvant and systemic chemotherapy, as well as in localizing relapse after treatment. In the new era of immunotherapy, 18F-FDG PET/CT may also be useful to monitor treatment efficacy as well as to detect immune-related adverse events. With the advent of artificial intelligence techniques such as radiomics and deep learning, these hybrid medical images can be mined for quantitative data, providing incremental value over current standard-of-care clinical and biological data. This approach has the potential to produce a major paradigm shift toward data-driven precision medicine with the ultimate goal of personalized medicine. In this review, we highlight current literature reporting the role of 18F-FDG PET in supporting personalized management decisions for patients with MIBC. Specific topics reviewed include the incremental value of 18F-FDG PET in prognostication, pre-operative planning, response assessment, prediction of recurrence, and diagnosing drug toxicity.

## Introduction

Bladder cancer (BC) is the 10th most common cancer worldwide, with approximately half a million new cases diagnosed globally and 200,000 related deaths per year (1). At diagnosis, 75% of the patients have non-muscle invasive BC whereas the remaining 25% have muscle invasive disease (MIBC). While non-muscle invasive BC is characterized by frequent recurrence (50–70%) but a relatively low propensity to progress (10–15%), MIBC has a poor prognosis with high rates of metastasis and 5-year survival <50% despite radical surgery (2).

The current standard treatment for MIBC is based on radical cystectomy (RC) with prior cisplatin-based neoadjuvant chemotherapy (NAC) in eligible patients (cT2-T4aN0M0) (3). For patients with more advanced stage disease or for those who recur after radical surgery, cisplatin-based combination chemotherapy remains the standard of care for first-line systemic treatment (3).

Recent advances in the field of immunotherapy are reshaping the therapeutic landscape for patients with BC. Specifically, immune checkpoints inhibitors (ICIs) have demonstrated promising results in both localized and metastatic settings (4–6). To date, five anti-PD(L)1 monoclonal antibodies have been approved by the US Food and Drug Administration in the second-line setting. This current shift in treatment strategy has created an unmet need to re-evaluate the clinical use of existing imaging techniques.

Although traditionally imaging of patients with BC has predominantly focused on CT (including CT urography) and MRI, 2-deoxy-2-(18F)fluoro-D-glucose (18F-FDG) positron emission tomography/computed tomography (PET/CT) may have the potential to offer additional diagnostic information due to its unique ability to image metabolism. Indeed, urothelial carcinoma, much like many other solid tumors, is characterized by alterations of glucose metabolism and overexpression of glucose transporters (GLUT-1 and GLUT-3) (7).

This review presents the most recent advances in 18F-FDG PET-guided personalized medicine in the context of MIBC. Through a review of the current literature, we present the potential clinical value of 18F-FDG PET/CT and PET/MRI for risk stratification at diagnosis, monitoring of treatment response, and detection of recurrence during follow-up.

## Risk Stratification at Diagnosis

Data supporting the potential role of 18F-FDG PET/CT for initial staging of MIBC are continuously increasing. Current data suggest that 18F-FDG PET/CT improves the detection of extravesical disease and can therefore substantially improve management decisions.

### Tumor Detection

2-Deoxy-2-(18F)fluoro-D-glucose positron emission tomography/computed tomography performance for tumor detection in the bladder is hampered by urinary excretion of 18F-FDG (8). Several studies have proposed using adapted protocols (hyperhydration, forced diuresis and refilling, or filling the bladder in a retrograde manner). Utilizing these techniques, authors report sensitivities between 50 and 96% (9–17). However, to date, 18F-FDG PET/CT has not be shown to improve primary tumor detection and staging, when compared to cystoscopy and morphological imaging alone performed with CT and especially MRI (18).

### Lymph Node Staging

Currently, the benefit of 18F-FDG PET/CT over contrast-enhanced CT (CECT) alone for lymph node (LN) staging remains controversial, since both modalities have excellent specificity but relatively poor sensitivity.

**FIGURE 1:**
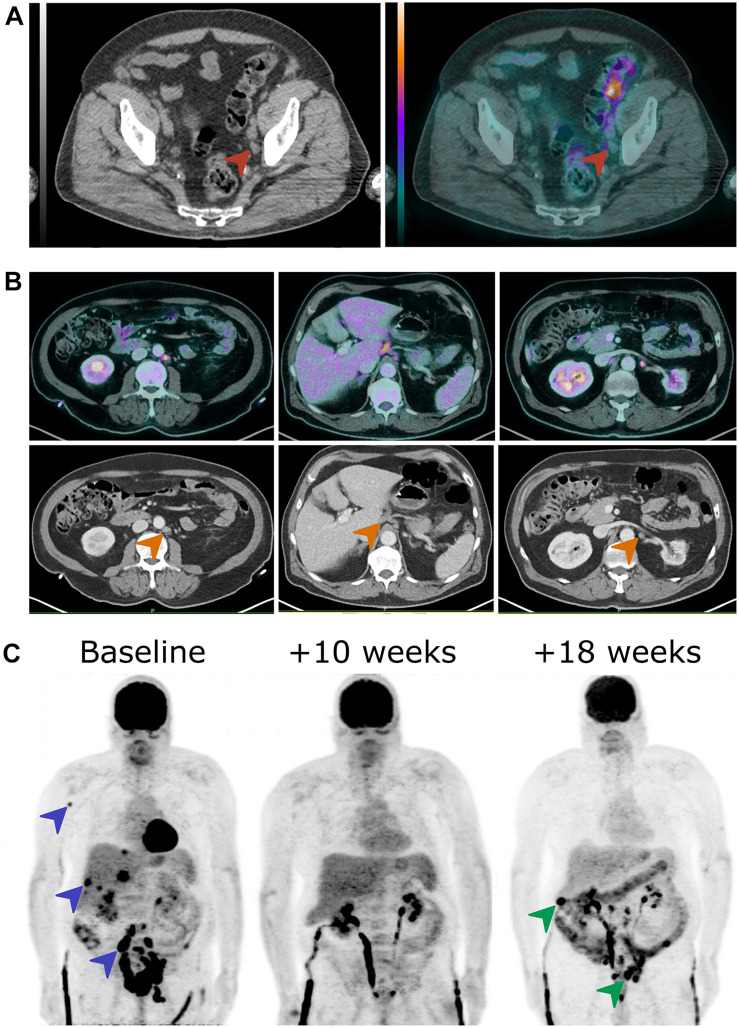
18F-FDG PET/CT images of three patients at different time points in the course of MIBC. **(A)** A 76-year-old man presented with an enlarged external iliac lymph node at initial staging without any 18F-FDG uptake (red arrowheads). The N0 status was confirmed by pathological examination of pelvic lymph node dissection sample. **(B)** A 67-year-old man was oligometastatic with one enlarged latero-aortic lymph node on CT at initial staging and 18F-FDG PET/CT revealed several other retroperitoneal lymph node metastases (orange arrowheads). **(C)** In a 62-year-old man who presented MIBC with osseous, hepatic, and nodal metastases at diagnosis (blue arrowheads), 18F-FDG PET/CT demonstrated complete metabolic response after three cycles of chemotherapy and then a locoregional and hepatic relapse after the fifth chemotherapy cycle (green arrowheads). 18F-FDG PET/CT, 2-deoxy-2-(18F)fluoro-D-glucose positron emission tomography/computed tomography; MIBC, muscle invasive bladder cancer.

A recent meta-analysis including 14 studies and 785 patients reported that the pooled sensitivity and specificity of 18F-FDG PET/CT for initial pelvic LN staging, in a per patient analysis, were 57% [95% CI 49–64%] and 92% [95% CI 87–95%], respectively (19). One major limitation of the current literature is the heterogeneous methodologies across published studies, notably regarding study designs, inclusion criteria, administration of NAC, injection–acquisition time in PET, the use of forced diuresis, the administration of contrast media for PET/CT, and interpretation criteria for both CECT and PET/CT (20). Studies comparing performance of CT to hybrid 18F-FDG PET/CT for pelvic LN staging with pathological analysis of extended pelvic LN dissection samples as a reference are presented in Table 1. Pooled sensitivities for CT and PET/CT are 38% [95% CI 29–47%] and 52% [95% CI 45–60%], respectively, while the pooled specificities are 91% [95% CI 88–94%] and 92% [95% CI 89–95%], respectively. Few studies have suggested a potential role for metabolic analysis by ruling out nodal disease in PET negative enlarged pelvic LNs (21, 22) (Figure 1A). Moreover, higher standardized uptake values (SUVmax) in LNs has been correlated with higher recurrence risk, independent of pathological findings (23).

**TABLE 1:** CT alone and 18F-FDG PET/CT performances for LN staging, with pathology as a gold standard, without preoperative chemotherapy.

First author	Year	CT criteria	PET/CT criteria	Sensitivity		Specificity		Accuracy	
				CT	PET/CT	CT	PET/CT	CT	PET/CT
Girard (22)	2019	SA > 8 mm	SUV_*max*_ > 4, and/or SA > 10 mm, and/or SUV_*max*_ > 2 and SA > 8 mm	7/17	8/17	38/44	42/44	45/61	50/61
Pichler (91)	2017	SA > 8 mm	SA > 10mm, and/or PET subjective analysis	5/11	7/11	54/59	52/59	59/70	59/70
Uttam (92)	2016	SA > 10 mm	SUV_*max*_ > 2.5	3/3	3/3	6/12	7/12	9/15	10/15
Jeong (93)	2015	SA > 10 mm or necrosis	SUV_*max*_ > 2.5	5/17	8/17	43/44	41/44	48/61	49/61
Aljabery (94)	2015	LA ≥ 10 mm	SUV_*max*_ > 2.5	7/17	7/17	33/37	32/37	40/54	39/54
Rouanne (95)	2014	SA > 10 mm	PET subjective analysis	NA	13/26	NA	74/76	NA	87/102
Goodfellow (26)	2014	SA > 8 mm	SA > 8 mm and/or PET subjective analysis	13/28	19/28	64/65	62/65	77/93	81/93
Hitier-Berthault (96)	2013	LA > 10 mm	PET subjective analysis	2/22	8/22	27/30	26/30	29/52	34/52
Swinnen (97)	2010	NA	PET subjective analysis	6/13	6/13	35/38	37/38	41/51	43/51
Kibel (33)	2009	NA	PET subjective analysis	NA	7/10	NA	30/32	NA	37/42
**Pooled [95% CI]**				**37.5% (48/128) [29.1–46.5%]**	**52.4% (86/164) [44.5–60.3%]**	**91.2% (300/329) [87.6–94.0%]**	**92.2% (403/437) [89.3–94.6%]**	**76.1% (348/457) [72.0–80.0%]**	**81.4% (489/601) [78.0–84.4%]**

*SA, short axis; LA, long axis; LN, lymph node; SUV_*max*_, maximum standardized uptake value; NA, not available.*

To our knowledge, there is only a single study of 18 patients comparing 18F-FDG PET/CT and conventional MRI for pelvic LN staging in MIBC. With pathology as the gold standard, authors reported sensitivity and specificity of 80% and 80% for MRI, and 93 and 88% for 18F-FDG PET/CT, respectively. There was no statistically significant difference. However, this study was limited by small sample size and the lack of multiparametric MRI sequences such as diffusion-weighted imaging (DWI) (24).

### Distant Metastatic Staging

Along with detecting nodal involvement, 18F-FDG PET/CT’s greatest strength is probably in detecting distant metastases during initial staging. In a patient-based analysis, 18F-FDG PET/CT sensitivity ranges between 54 and 87%, while specificity ranges between 90 and 97% for the detection of distant metastases from BC (15, 25–27). One study reported 18F-FDG PET/CT to be more sensitive than CT alone for detection of distant metastases, with sensitivities of 54 versus 41%, respectively. Both modalities showed similar very high specificities of 97 and 98%, respectively (26). Thus, 18F-FDG PET/CT revealed more lesions suspected to be metastasis or second primary cancer than conventional imaging (26, 28–30). 18F-FDG PET/CT changed management over conventional imaging in a range of 18–68% of patients (25, 29, 31, 32), and resulted in less additional tests in 70% of patients (25). The presence of FDG-avid regional LNs or extra pelvic lesions was an independent predictor of overall survival (28, 33), whereas it was not statistically significant for the counterpart conventional CECT findings (28).

In conclusion, the current literature suggests that 18F-FDG PET/CT provides an incremental value for nodal and distant staging in MIBC at initial diagnosis. However, due to its significant cost for healthcare systems (34), prospective studies with high evidence level are still needed before its use can be formally adopted into consensus guidelines and recommendations (3, 18, 35, 36). In support of its role in distant staging, a recent consensus statement revealed that 18F-FDG PET/CT is the imaging modality of choice to avoid over-treatment in oligometastatic patients with an agreement of 88% of participants (37) (Figure 1B).

### Metabolic Prognostic Factors

Several prognostic biomarkers can be extracted from 18F-FDG PET using routine clinical workstations. The mainstream prognostic imaging biomarkers are baseline metabolic tumor burden (TMTV), baseline total lesion glycolysis (TLG), and SUVmax. All have been associated with overall survival in a wide range of cancers and treatments (38–40). With the paradigm shift of immunotherapy, authors have evaluated the potential role of appraising host metabolism in lymphoid tissues such as the bone marrow. They have demonstrated that increased bone marrow metabolism is associated with shorter overall survival and systemic immune suppression (41, 42).

In patients with advanced BC, such prognostic factors have not been reported as of yet. Additionally, there are no studies demonstrating that these biomarkers can predict response to chemotherapy or ICIs. In a proof of concept study, Chen et al. suggested that higher 18F-FDG uptake by BC may be associated with elevated PD-1/PD-L1 expression, potentially guiding the decision to select patients for ICIs (43, 44).

## Response Evaluation

Based on a limited number of studies, 18F-FDG PET/CT appears to be a valuable tool for monitoring response to chemotherapy both in the neoadjuvant and metastatic settings. While the performance of 18F-FDG PET/CT in evaluating tumor sensitivity to immunotherapy has been demonstrated in several solid malignancies, it remains to be established for MIBC.

### Neoadjuvant or Induction Treatment

There have been a few studies that have focused on the performance of 18F-FDG PET/CT in monitoring the response of BC to NAC. In terms of primary bladder tumor evaluation after NAC, 18F-FDG PET/CT has demonstrated 75% sensitivity and 90% specificity in identifying patients with complete pathologic response (45). After induction chemotherapy in pelvic LN metastatic patients, responders were distinguished from non-responders with a sensitivity of 83–100% and a specificity of 67–94% with 18F-FDG PET/CT, compared to 88 and 33%, respectively, with conventional CECT. Complete responders were correctly identified with 67–75% sensitivity and 46–90% specificity with 18F-FDG PET/CT, compared to 64 and 60% with CECT, respectively (45–47). These results suggest that 18F-FDG PET/CT may be useful to assess response to neoadjuvant or induction chemotherapy, but is of limited interest to select patients for RC due to low predictive value in detecting residual LN involvement (47, 48).

To our knowledge, there is no published study investigating the role of 18F-FDG PET/CT in evaluating response after preoperative immunotherapy.

### Metastatic Bc: Chemotherapy

2-Deoxy-2-(18F)fluoro-D-glucose positron emission tomography/computed tomography is useful for evaluating response to systemic chemotherapy (Figure 1C). In one study, 18F-FDG PET/CT using the European Organization for Research and Treatment of Cancer (EORTC) criteria outperformed CT interpretation alone based on Response Criteria in Solid Tumors (RECIST) criteria for the prediction of response to first-line systemic chemotherapy (cisplatin and gemcitabine) (49). Furthermore, early response assessment using 18F-FDG PET/CT predicted progression-free survival and overall survival after two cycles of combination of methotrexate, vinblastine, doxorubicin, and cisplatin (MVAC) in first-line metastatic chemotherapy (50).

### Metastatic Bc: Immunotherapy

The long-term benefit of first-line immunotherapy compared to carboplatin-based chemotherapy was recently reported in patients with metastatic or locally advanced urothelial carcinoma (5). In this new era of immuno-oncology, the treatment paradigm is shifting toward restoring tumor elimination by the immune system. This new treatment paradigm has introduced novel patterns of response and progression, such as pseudo-progression and hyperprogression, which have been observed in a wide range of cancers, particularly using 18F-FDG PET/CT (51).

Pseudo-progression is a well-described novel immune-related pattern of response. Pseudo-progression is defined as a transient increase followed by a decrease in apparent total tumor burden. Its incidence is highly variable between studies, ranging between 2 and 10%, depending on tumor type, treatment, and patients (52, 53). Pseudo-progression incidence rates have been reported as ranging between 1.5 and 17% of patients with advanced urothelial carcinoma on immunotherapy (54). Thus, the majority of apparent early progressive disease visualized on 18F-FDG PET/CT represents true progression rather than pseudo-progression (55).

Hyperprogression is defined as a rapid increase in tumor growth rate (≥2-fold) compared to the expected growth rate in cancer patients treated with Anti-PD-1/PD-L1 agent. Hyperprogression occurs with an incidence of 9% in solid tumors and is associated with a poor outcome (56). The definition of hyperprogression on 18F-FDG PET/CT is currently controversial since metrics differ between institutions (57).

Immune-related adverse events (IrAEs) are well-known side effects of ICIs that can involve almost all organs. IrAEs in patients with MIBC treated with ICIs occur in up to 23% of patients, similar to patients with other solid malignancies (6). 2-Deoxy-2-(18F)fluoro-D-glucose positron emission tomography/computed tomography performed during the treatment for restaging and/or response assessment can also reveal a wide range of IrAEs, (e.g., sarcoidosis-like syndrome, thyroiditis, hypophysitis, enterocolitis, interstitial pneumonitis, pancreatitis, and arthritis) with an accuracy of 83% (58, 59). Authors recently reported that shared genetic factors impact risk for IrAEs and survival on immunotherapy in BC (60).

To date, there have been no studies investigating the specific role of 18F-FDG PET/CT in evaluating the response of advanced BC to immunotherapy.

## Recurrent Disease

Few published studies have evaluated the diagnostic performance of 18F-FDG PET/CT in detecting relapse of BC after systemic chemotherapy and/or RC (Figure 1C). Sensitivity is reported to be between 87 and 92% and specificity between 83 and 94%, with a significant change in the management of up to 40% of patients compared to conventional imaging alone (61–63). Alongi et al. reported SUVmax > 6 and TLG > 8.5 of recurrent bladder tumors as the most significant predictors of 2-year progression-free survival (61).

## Technical Considerations, New Tracers, and Devices

Although several studies have demonstrated the clinical utility of 18F-FDG PET/CT throughout the management of patients with BC, technical caveats should be considered.

### Pet Technology

Over the past decade the gradual spread of 3D PET equipped with time-of-flight technology and using iterative reconstruction algorithms including point-spread-function correction has significatively improved image quality. More specifically, these recently developed technologies have enhanced detectability of subcentimeter foci of disease (64) and therefore may significantly influence performances of PET/CT in regard to nodal disease (65).

### Rationale for Diuretic Protocols

Although detection and characterization of primary bladder tumors is not the aim of 18F-FDG PET/CT, adapted protocols are of interest in terms of improving LN staging in pelvic malignancies by lowering artifacts caused by concentrated urinary radioactivity (66). Due to its diagnostic performance with respect to nodal disease, 18F-FDG PET/CT protocols for BC should integrate forced diuresis (with 20 mg to 40 mg of furosemide intravenously) and oral hyperhydration (1.5–2 L) in everyday clinical practice (10, 16). Supplementary delayed pelvic images may be helpful in selected patients with inconclusive standard images.

### Early Dynamic Acquisitions

In an effort to improve tumor conspicuity through an increase of tumor-to-urine SUVmax ratio, few authors have investigated the utility of early dynamic acquisitions, before radioactive urine has had a chance to fill the bladder (13, 67, 68). In these proof-of-concept studies, the authors suggested that such dynamic acquisitions might improve tumor detection and staging; however, the impact on LN staging was not evaluated.

### Other Pet Tracers

In an effort to overcome the limitations of excreted urinary 18F-FDG in the setting of BC and pelvic LN evaluation, additional PET tracers have been studied. These studies have not demonstrated sufficient improvement to justify implementation of these tracers in everyday practice.

11C-Acetate PET/CT has been investigated in small cohorts. It has not shown significantly different results compared to MRI and CECT for tumor detection and LN staging (69–71).

In BC patients before cystectomy, 11C-Choline PET/CT detected pelvic LN involvement with an accuracy of 81% (sensitivity of 90% and specificity of 71%) (72). In an intra-patient comparison, 11C-choline PET/CT appeared to have no significant advantage compared to 18F-FDG PET/CT (73). In two studies comparing 11C-Choline PET/CT to conventional imaging for LN staging, sensitivity was of 42–58% and specificity was 66–84% for 11C-Choline PET/CT, compared to 14–75% and 56–90%, respectively, with CECT alone (74, 75). At initial staging, 11C-Choline PET/CT was not able to significantly predict overall survival or cancer-specific death (76). Graziani et al. studied the performance of 11C-Choline PET/CT in detection BC relapse. The authors reported a sensitivity of 67% and a specificity of 85% for local relapse and a sensitivity of 90% and specificity of 92% for distant relapse (77). These results are in line with the performance of 18F-FDG PET/CT.

Regarding the investigation of bone metastases, 18F-sodium fluoride PET/CT has been shown to reveal more bone metastases than standard bone scintigraphy (78) or 18F-FDG PET/CT (79).

### Rationale for Pet/Mri

Thanks to its superior contrast resolution, MRI is currently the most effective non-invasive imaging modality for local staging of BC, with an accuracy between 92 and 98% for detection of muscle invasion (18, 80). In spite of this contrast resolution, MRI’s anatomical sequences (T2 weighted, T1 weighted) have not been shown to be superior to CECT in detecting LN involvement. Combining functional sequences such as DWI and dynamic contrast enhancement (DCE) still results in a relatively low sensitivity (80). Thus, MRI and PET complement one another for the staging of BC; the former is useful for local staging, the latter for distant metastasis detection, while together they may be synergistic for revealing pelvic LN involvement.

To date, only small studies have been published investigating the role of PET/MRI in MIBC. In a prospective pilot study including 24 patients, Rosenkrantz et al. highlighted the potential interest of hybrid 18F-FDG PET/MRI over MRI alone including DWI, especially to detect pelvic LN involvement with an accuracy of 95% for PET/MRI versus 76% for MRI alone, as well as non-nodal extravesical pelvic involvement with an accuracy of 100 versus 91%, respectively (81). In contrast, another recently published pilot study investigating PET/MRI in MIBC patients after NAC (*N* = 18, with LN involvement in only three patients) showed a sensitivity of 80% and a specificity of 56% for detection of the primary tumor, and 0% and 100%, respectively, for detection of LN involvement (82).

### Rationale for Artificial Intelligence

Recent advances in artificial intelligence (AI) techniques are able to harness routine PET images into data that can be mined to extract imaging biomarkers for purposes of guiding precision medicine for BC. The term AI encompasses distinct fields such as deep learning, and radiomics, which go beyond the scope of this review. AI can be leveraged on clinical data, molecular and genetic biomarkers, and imaging for several narrow tasks in BC. While there are currently no studies published on the use of AI on 18F-FDG PET images for BC, promising results of its application using CT and MRI images have been reported in terms of predicting the depth of invasion of the primary tumor (83), grade (84), local and systemic staging (85), and assessment of treatment response (86). Additionally, AI based on PET/CT images has been used in other malignancies to predict nodal disease (87), risk stratification (88), treatment response (89), and patient outcomes (90). The main advantages of using AI is its potential reproducibility, as compared to the inherently subjective interpretation of medical images by physicians as well as the ability to harness large quantities of data that may escape the pattern recognition abilities of humans.

## Conclusion and Future Directions

There is strong and still evolving evidence supporting the utility of 18F-FDG PET/CT in the management of MIBC. 18F-FDG PET/CT appears to outperform and/or complement conventional imaging techniques for several tasks. Current data support the idea that 18F-FDG PET/CT may help to select the most efficacious treatment for each patient at each step of MIBC management. There is some agreement among the medical community that 18F-FDG PET/CT is relevant to guide management at initial staging in patients considered as oligometastatic by conventional imaging, such as patients with enlarged pelvic LN. However, to date, prospective studies with high level of evidence are lacking in order to allow the systematic adoption of 18F-FDG PET/CT in structured guidelines. With the advent of AI, the broad range of clinical, biological, anatomical, and metabolic data may be harnessed in order to lead to improved precision medicine.

## Author Contributions

AG, LD, and MR designed the review article. AG, HVR, MR, HS, and LD performed the literature review and wrote and edited the manuscript. J-FG, OD, P-YS, and HS critically revised the manuscript. All authors contributed to the article and approved the submitted version.

## Conflict of Interest

The authors declare that the research was conducted in the absence of any commercial or financial relationships that could be construed as a potential conflict of interest.
